# Novel Means for Photoprotection

**DOI:** 10.3389/fmed.2018.00162

**Published:** 2018-05-29

**Authors:** Kevin Sondenheimer, Jean Krutmann

**Affiliations:** ^1^IUF – Leibniz Research Institute for Environmental Medicine, Düsseldorf, Germany; ^2^Medical Faculty, University of Düsseldorf, Düsseldorf, Germany

**Keywords:** photoprotection, visible light, blue light, red light, infrared, Ultraviolet-B, Ultraviolet-A, DNA repair enzyme

## Abstract

Due to changes in human lifestyle (expanded sunbathing, the use of solaria, etc.) and, most importantly, increasing lifetime and thus higher cumulative exposure to solar radiation, skin aging and skin cancer have become major health issues. As a consequence effective photoprotection is of outmost importance to humans. In this regard a lot has been learned in the past about the cellular and molecular basis underlying ultraviolet (UV) radiation-induced skin damage and, based on this knowledge, numerous skin protective approaches including organic and inorganic UV-filters, but also topically applicable antioxidants, DNA repair enzymes and compatible solutes as well as oral photoprotective strategies based on nutritional supplements have been developed. A new aspect is here that sun protection of human skin might even be possible after solar radiation-induced skin damage has occurred. A second, very important development was prompted by the discovery that also wavelengths beyond the UV spectrum can damage human skin. These include the blue light region of visible light (VIS) as well as the near infrared range (IRA) and corresponding sunprotection strategies have thus recently been or are still being developed. In this article we will provide a state of the art summary of these two novel developments and, at the end, we will also critically discuss strengths and weaknesses of the current attempts, which mainly focus on the prevention of skin damage by selected wavelengths but greatly ignore the possibility that wavelengths might interfere with each other. Such combined effects, however, need to be taken into account if photoprotection of human skin is intended to be global in nature.

## Introduction

Reflected and filtered by the atmosphere only a part of sun light reaches the surface of the earth. This radiation can induce harmful effects on human skin including sunburn, immunosuppression, photoaging and skin cancer. It is generally thought that high-energetic Ultraviolet radiation (UVB, 280–315 nm and UVA, 316–400 nm) is mainly responsible for these adverse effects. As a consequence, traditional photoprotection of human skin was restricted to protection against UV-rays. More recently, this view has been changed by an increasing number of independent scientific reports indicating that (i) also wavelengths of the solar spectrum beyond UV radiation, VIS (400–770 nm) and near infrared radiation (IRA, 771–1,440 nm), can damage human skin and (ii) that there is growing evidence that photoprotection is also possible after sun-induced skin damage has occurred. Here, we will summarize these novel developments and will critically discuss strengths and weaknesses of existing approaches. We will conclude by providing our view on upcoming challenges, which we believe, will further improve the performance and efficacy of sun protection of human skin.

## Protection against IRA

IRA is the major component of natural sunlight and approximately 30% of the total solar energy reaching the earth's surface is within the IRA range. Traditionally, photodermatology focused mainly on physiological, pathophysiological and therapeutical effects of UVB- and UVA-radiation whereas wavelengths in the IRA range have long been ignored. In recent years, however, the number of studies addressing IRA-induced skin damage increased and today, it is generally accepted that wavelengths in this range similar to UV radiation (UVR) can induce skin damage. Skin damage caused by IRA radiation mainly manifests as perturbation of extracellular matrix homoeostasis by degrading dermal connective tissue which clinically presents as wrinkle formation. These findings have been recently reviewed in great detail and we will therefore only mention important key studies for the purpose of this review.

The impact of IRA on human skin is best illustrated by Calles et al. who showed that approximately 600 genes are IRA responsive in human dermal fibroblasts. By functional clustering these identified genes could be assigned to groups involved in extracellular matrix homeostasis, apoptosis, cell growth and cellular stress response (1). In line, additional studies addressing IRA-induced skin damage reported of an increased expression of matrix degrading enzymes such as MMP-1 and MMP-9 (matrix metalloproteinase-1/9), along with a decreased collagen production (2–4). Some of these studies were criticized by using irradiation doses, which exceed physiological doses a human being is usually exposed to natural sunlight. Of note, however, similar effects have been reported using low or moderate doses of IRA *in vitro* (5). More importantly, these findings were underlined by a recent study of Cho et al. in which natural sunlight was filtered to allow for study of IRA and heat (4). In aggregate, all these studies show that IRA-irradiation can cause wrinkle formation by enhancing the expression of matrix degrading enzymes.

An obvious approach for an effective protection against IRA-induced skin damage would be the use of physical or chemical filters similar to classical UV protective sunscreens. Regularly used compounds, however, have not been shown to possess significant IRA-filtering capacities (6). Although, inorganic pigments with IR-reflecting properties are well known, e.g., coloring pigments used as roof coatings, these substances have a major disadvantage because they would be visible to the consumer after topical application (7). Alternatives that might cause less or no compliance problems could be formulations containing fumed silica which disperse and block infrared radiation (8). Of note, many studies provide evidence that IRA-induced skin damage is mediated around the generation of ROS (reactive oxygen species) (2, 9, 10). Therefore, photoprotection of human skin against wavelengths in this range now mainly involves topically applied antioxidants. In this regard, it is important to emphasize that IRA photoprotection requires specific antioxidants as it could be shown in a target-driven *in vitro* screen in primary human skin fibroblasts. By using IRA-induced MMP-1 mRNA expression as a read-out model certain polyphenols and vitamins could be identified as effective compounds. This was confirmed *in vivo* by topical application of an antioxidant mixture 20 min before IRA-exposure (2). This study actually prompted the development of topical sunscreen products with an efficient protection against IRA, which were first launched in Germany. In the meantime, additional studies have been performed pointing out the necessity of sunscreens, which offer an efficient protection against IRA (3, 10–12). Today, IRA photoprotection is no longer limited to sunscreens but similar to UV protection it may be found in daily skin care products as well (13). Although antioxidants are less potent in preventing sunburn in contrast to classical sunscreens (14) appropriate concentrations of orally administered antioxidants might represent an alternative. These compounds have the advantage that in contrast to topical antioxidants, which might poorly penetrate into the skin and be unstable, the entire skin surface is protected without being affected by washing, perspiration or rubbing.

A persistent major challenge for the development of antioxidants for IRA protection of human skin results from the fact that no standardized *in vitro* or *in vivo* test exists to validate photoprotective properties. Whereas, erythema and pigmentation represent easy to measure biological endpoints for UVA or UVB sunscreens, no endpoints have been identified for IRA, which can be measured non-invasively in human skin. Therefore, we recommend to use IRA-induced MMP-1 mRNA expression in human dermal fibroblasts to screen selected antioxidants in a first step. In a second step, a clinical study should be performed in which complete sunscreen products containing candidate molecules which are proven to be effective *in vitro* are tested as formulations to assess their potential to inhibit IRA-induced MMP-1 mRNA expression in human *ex vivo* skin models or, ideally, *in vivo* in human skin.

## Protection against VIS

Visible light is defined as part of the electromagnetic spectrum that ranges from violet (400 nm) to profound red (770 nm). In contrast to numerous studies which addressed IRA-induced skin damage the number of studies centered around VIS and skin is limited to a few.

Zastrow et al. reported of an increased radical formation analyzed by electron spin resonance in *ex vivo* human skin after irradiation not only with UVR and IRA but also in the VIS range (15). This could be confirmed and extended by a second study using Electron paramagnetic resonance spectrophotometry *ex vivo* and *in vivo* (16). In human epidermis models, VIS is able to induce MMP-1 as well as TNF-α (tumor necrosis factor alpha) mRNA expression in keratinocytes by an increased production of ROS. This increased radical formation was confirmed in *in vivo* human skin by Raman spectroscopy (17). Direct biological consequences of VIS-irradiation on human skin were first shown by Pathak et al. These authors provided evidence that wavelengths in the VIS/long-wavelength UV range at physiological relevant doses can cause pigmentation *in vivo* in human skin (18, 19). This could be confirmed by a recent study using an artificial irradiation device without contaminating UVR rays and an emission spectrum mainly containing wavelengths between 400 and 800 nm. Of note, increased pigmentation occurred only in darker pigmented skin types ≥III according to Fitzpatrick scale (20). Similar results were observed and could be extended in an independent study in which a marked and prolonged skin pigmentation was induced by blue-violet light in a dose dependent manner, whereas red light did not induce any pigmentation. Compared to UVB-induced hyperpigmentation, blue-violet light induced a more pronounced pigmentation that lasted up to 3 months and histological stainings revealed decreased levels of p53 and necrosis of keratinocytes (21). The absence of p53 activation in pigmentation after blue light irradiation suggests mechanisms, which are different from those known to be involved in the response to UVB. Accordingly, a recent study showed that melanocytes are directly affected by blue light and increase melanin synthesis in response to blue light-induced activation of Opsin-3 receptors on their surface. This mechanism is calcium dependent and involves a kinase-dependent signaling cascade leading to the activation of the transcription factor MITF (Microphthalmia-associated transcription factor) and further to an increased expression of melanogenesis related tyrosinase and dopachrome tautomerase. These enzymes form a complex which is mainly induced in dark skin melanocytes and leads to sustained tyrosinase activity (22). There is also indirect evidence that exposure to VIS can worsen melasma. An iron oxide containing sunscreen providing protection against UVB/UVA plus VIS proved to be superior to a control sunscreen with identical UVB/UVA but without VIS protection in the prevention of melasma relapse (23). There is currently no evidence that VIS can cause health effects beyond skin hyperpigmentation/melasma. In particular, VIS has not been shown to cause wrinkle formation.

Of note, ROS formation and accumulation is a key mechanism for the expression of keratinocyte-derived cytokines, but VIS-induced pigmentation does not involve ROS formation and cannot be targeted by antioxidants. Therefore, to the best of our knowledge, protection against VIS in terms of pigmentation may only be provided by scattering or reflecting VIS in the blue-violet range. The absorption or reflection range of commonly used inorganic sunscreen agents like iron oxide, titanium dioxide or zinc oxide, ranges from UVR to VIS but greatly depends on the particle size. Only optically opaque sunscreen formulations containing inorganic pigments are able to reflect and scatter VIS (24) but these compounds are water-insoluble and leave a white or tinted coating on skin which is unacceptable for most costumers. This was confirmed by a study, which assessed the protective efficacy of several sunscreens containing titanium dioxide and iron oxide against VIS-induced pigmentation in darker pigmented skin types. Here, pretreatment of skin with a VIS-filtering sunscreen based on inorganic compounds reduced VIS-induced pigmentation up to 5 days after exposure (25).

However, in order to develop highly efficient sunscreens against VIS which are also consumer compatible further basic research is clearly needed.

## Photoprotection after sun exposure

A completely new approach is the concept that photoprotection is also possible even after skin damage has occurred. The main goal of such protection strategies is to support or enhance DNA repair by supplying biological active enzymes imbedded in an absorbable formulation.

This can be achieved by the presence of DNA repair enzymes in after-sun lotions or creams, which has been shown to work in a study of Stege at al. Topical treatment of human skin with liposomes containing active photolyase and subsequent exposure to photoreactivating radiation led to an enhanced removal of UVB-induced cyclobutane pyrimidine dimers, diminished erythema and sunburn-cell formation as well as suppresses UV-induced expression of ICAM-1 (intercellular adhesion molecule-1), an enzyme which is required for inflammatory immune response in the epidermis (26). In another clinical study, Wolf and co-workers have demonstrated that liposomes containing the DNA repair enzyme T4 endonuclease prevent the UV-induced upregulation of immunosuppressive cytokines in patients with a history of skin cancer. The repair enzyme penetrated into the human skin and was located in keratinocytes and epidermal Langerhans' cells (27). Similar formulations were also tested in several clinical studies on the prevention of actinic keratosis (AK). By treatment of the precancerous field of AK with a medical device containing conventional UV-filters and biological active photolyase a significant general improvement of the skin was observed and an over-expression of fundamental processes related to tissue reconstruction, e.g., cell communication, signaling and adhesion could be demonstrated (28). In line with these observations, a 9-months randomized clinical study analyzed the impact of a sunscreen containing photolyase on patients with AK after photodynamic therapy. Compared with a conventional sunscreen the daily application of sunscreen plus photolyase was associated with a significant prevention of new AK lesions. During treatment, no additional phototherapy was required in the photolyase group, whereas newly AK lesions developed in the group receiving sunscreen only (29). This strongly indicates that DNA repair enzymes used in sunscreens are able to prevent the development of AK's in human skin.

## Outlook

Traditionally, the majority of photodermatologic studies analyzed each wavelength range, i.e., UVB, UVA, VIS or IRA-induced biological effects on human skin, separately. However, human skin is naturally exposed to all of these wavelengths simultaneously, and it is conceivable to assume that interactions or interferences between these wavelengths exist that may fundamentally influence the overall biological response and therefore are of utmost importance for the development and improvement of photoprotection.

Support of this concept was first provided by Schieke et al. who investigated the molecular crosstalk of UVA and UVB on activation of MAPK (mitogen-activated protein kinase). In a first step, the activation pattern after single irradiation with UVA or UVB was analyzed resulting in a UVA-induced modest and transient phosphorylation of ERK 1/2 (extracellular signal-regulated kinase-1/2), 15–30 min after exposure whereas UVB irradiation caused a strong and immediate activation that lasted up to 1 h. Activation of p38 and JNK 1/2 (c-jun N-terminal kinases-1/2) was only slightly enhanced after single irradiation. A different pattern was observed if keratinocytes were sequentially exposed to UVA and UVB. In this case, p38 and JNK 1/2 phosphorylation were enhanced, but the UVB-induced immediate activation of ERK 1/2 was prevented, regardless of the irradiation sequence (30). This study has shown that a molecular crosstalk of UVB and UVA exist which has been observed on level on MAPK signaling and may represent and evolutionary conserved defense strategy of human skin cells to respond to solar radiation-induced stress. A second study was published in 2007 demonstrating that apoptosis after simultaneous irradiation with UVB+UVA (solar simulated UVR) in comparison to single UVB is ameliorated in a UVA dose dependent manner *in vivo*. Here, histological analysis of sunburn cell formation and caspase-3 activation revealed that apoptosis in mice can be reduced up to 50% after 24 h post-exposure to 3MEdD (minimal edematous dose) of solar simulated UVR. This effect is probably mediated by increased heme oxygenase activity, an enzyme which plays an important role in the protection against oxidative stress in human skin (31). Of note, different ratios of UVA/UVB, which were used for irradiation in this study, are of high physiological relevance concerning the altering emission spectrum of the sun, which is strongly affected by daytime, weather conditions and the season. In addition to apoptosis, also UVB-induced immunosuppression was shown to be ameliorated after irradiation with solar simulated UVR *in vivo*. Interleukin-6 which is released after UVB could be identified as an essential factor of the UVA-mediated protective effect on the immune response (32). There is also evidence that crosstalk signaling may exist for UVB and IRA radiation, although in most of these cases the sequence of irradiation (first UVB subsequently IRA vs. first IRA subsequently UVB) fundamentally influenced the biological response (reviewed in Grether-Beck et al.). In aggregate, these results strongly indicate that simultaneous UVB+UVA irradiation causes a third biological response, which differs from single UVB or UVA exposure, and even more important cannot be explained by a simple addition of biological effects.

These examples emphasize the need for detailed simultaneous irradiation studies targeting the analysis of the relative contribution of each wavelength to the entire biological effect of solar radiation-induced skin damage. Considering that human skin has perfectly adapted to natural sunlight during evolution then the exposure to the complete solar spectrum provides an optimized stress response with the overarching goal to limit skin damage as much as possible. Therefore, studies which use irradiation protocols to single wavelengths only or merely sequentially add two or more wavelengths ranges may lead to results which are of limited physiological relevance or are completely misleading. We therefore believe that this issue can be best assessed by the development of a novel irradiation device which (i) allows simultaneous irradiation with UVB, UVA, VIS and IRA at physiologically relevant doses *in vitro* and *in vivo*, (ii) but to selectively dim off specific wavelengths areas to understand their relative contribution to the entire biological effect and (iii) to change continuously the intensity of each installed lamp especially in the range of UVB and UVA to simulate variations of the emission spectrum as it is the case for natural sunlight during daytime or seasons. As a consequence, we have recently built this irradiation device, which is currently used in *in vitro, ex vivo* and *in vivo* studies to better understand the interaction of different wavelengths present in natural sunlight.

## Author contributions

KS: literature research, writing and drawing of figures; JK: literature research and writing. All authors read and approved the final version of the manuscript.

### Conflict of interest statement

JK served as a consultant to companies that develop cosmetic products for photoprotection, and the IUF has received financial support from such companies to assess the efficacy of cosmetic and sunscreen products in protection against infrared radiation and visible light. The other author declares that the research was conducted in the absence of any commercial or financial relationships that could be construed as a potential conflict of interest.
